# Adolescents' Experiences of Weight‐Related Bullying in the School Setting—A Qualitative Study

**DOI:** 10.1111/apa.70652

**Published:** 2026-06-13

**Authors:** Emelie Andersson, Nicole Mpatsiaris Riboe, Kajsa Järvholm

**Affiliations:** ^1^ Department of Psychology Lund University Lund Sweden; ^2^ Childhood Obesity Unit Skåne University Hospital Malmö Sweden

**Keywords:** children, obesity, stigma, victimisation

## Abstract

**Aim:**

Obesity is a stigmatised disease, and weight‐related bullying is the most common form of victimisation. This study aimed to provide a nuanced understanding of adolescents’ experiences of such victimisation and related interventions.

**Methods:**

Data were collected between April and October 2025 through semi‐structured interviews and analysed using reflexive thematic analysis. Participants were Swedish adolescents aged 12–19 years, primarily recruited from obesity units.

**Results:**

The analysis generated three main themes. *My body is the problem* reflects how weight‐related bullying is experienced in everyday contexts and how adolescents encounter pervasive messages devaluing their bodies and self‐worth. *When the school turns a blind eye* illustrates how the school fails to protect and at times becomes part of the problem. *I'm ruined for life* captures how bullying erodes trust, self‐confidence, and optimism about the future.

**Conclusion:**

Adolescents report that weight‐related bullying has substantial negative effects on both their current and future well‐being. They also perceive that schools do not take such bullying seriously enough. Healthcare providers should routinely assess whether adolescents with obesity experience weight‐related bullying and respond appropriately. Schools must recognise weight‐related bullying as a pervasive issue and implement strategies that do not place responsibility on the victim.

## Key Points

- Weight‐related bullying is common among adolescents with obesity, yet little is known about how they themselves experience it and how they perceive school responses.
- The study found that adolescents encounter both subtle and overt weight‐related bullying that harms their well‐being in the present and shapes their expectations for the future.
- Clinicians should routinely assess bullying, and schools need strategies that effectively address perpetrators rather than shifting responsibility to victims.

**Abbreviations**
EA: Emelie Andersson
KJ: Kajsa Järvholm
NMR: Nicole Mpatsiaris Riboe

## 1 Introduction

The proportion of children and adolescents with overweight or obesity is increasing [1]. Obesity is a highly stigmatised disease, and international studies have shown that weight‐related bullying is the most common form of victimisation among girls and boys, with 50% of girls with obesity reporting having been bullied during the last 30 days [2]. In a study including treatment‐seeking adolescents with obesity, 64% reported being exposed to weight‐related bullying in school, and among those exposed, 78% reported that the weight‐related bullying had been going on for at least one year [3].

Being bullied in school is a serious threat to young people's development, as it is associated with increased risk of mental health problems, including suicidal behaviour and substance use [4]. Adolescents exposed to weight‐related bullying are more likely to report lower self‐esteem, more symptoms of anxiety and depression, suicidal thoughts, and attempts than youth who were not bullied [5, 6]. They are also more likely to develop eating disorders, especially bulimia and binge eating disorder [7]. One study showed that adolescents exposed to weight‐related bullying were up to five times more likely than their non‐bullied peers to be absent from school [8]. Furthermore, exposed adolescents have been found to have more severe obesity 15 years later compared to unexposed adolescents with obesity [9].

Despite being common and having serious negative consequences, weight‐related bullying has received limited attention in research. Previous qualitative research has shown that adolescents living with obesity frequently experience stigma, bullying, and relational insecurity [10]. Weight‐related bullying has been described as recurring over several years, with many adolescents coping through avoidant strategies such as isolation and self‐harm [11, 12]. While previous qualitative research offers important insights, weight‐related bullying has typically been addressed as part of a broader focus, in non‐school contexts, or in relation to other factors such as coping strategies [12]. To achieve a nuanced understanding of the phenomenon, it is essential to thoroughly examine the lived experiences of adolescents who have been subjected to weight‐related bullying. The aim of this study was to explore the experiences of adolescents who were or had been the subjects of weight‐related bullying in the school context and to give voice to their unique perspective. We also aimed at exploring the adolescents' perceptions of the school's strategies for addressing weight‐related bullying, and the measures they wished the school had implemented.

## 2 Methods

Participants were recruited through purposive sampling between April and October 2025. Inclusion criteria were age 12–19 years, current or previous experiences of weight‐related bullying, current or previous overweight or obesity, and the ability to conduct the interview in Swedish. Information about the study was disseminated in both written form (via posted notices) and orally through multiple paediatric obesity units across Sweden, as well as via the patient association Obesity Sweden's social media channels and website.

In total, 13 adolescents (ten girls and three boys) were included and interviewed. All but one were recruited via obesity clinics, and all had obesity. The mean age was 15.5 years (range 12–19 years). Participants were informed that the project aims to develop a deeper understanding of adolescents' experiences of weight‐related bullying, with the long‐term goal of improving the ability of schools, student health services, and society to identify, address, and respond to such bullying.

To gain an in‐depth understanding of adolescents' experiences of weight‐related bullying, data were collected using semi‐structured interviews. The development of the interview guide was informed by the study aim, research questions, existing literature on weight‐related bullying, and clinical experience working with adolescents with obesity. No participant involvement was included in the development of the guide. The format allowed participants to speak freely about the topics in relation to broad questions such as “Did/does anyone know that you were/are being bullied?” and “What kind of support have you received from the school when experiencing weight‐related bullying?”. The interviewer could ask follow‐up questions. Following the initial interview, the interview guide was deemed appropriate and required no modifications.

All interviews were conducted between May 7 and October 15, 2025, by either EA or NMR (both females, then in the final stage of clinical psychology training and now MSc graduates, with no previous contact or clinical relationship with participants). EA and NMR found the interviews emotionally heavy, reflecting the sensitive and sometimes painful experiences shared by participants. Eight participants were interviewed digitally, and five were interviewed at a paediatric obesity clinic. While both digital and in‐person interviews enabled meaningful and in‐depth conversations, EA and NMR noticed that the in‐person format enabled a sense of physical presence and non‐verbal responsiveness that was difficult to fully achieve digitally. The duration of interviews ranged from 13 to 51 min, with an average of 30 min. The length of the interviews varied naturally across participants: Some adolescents elaborated extensively on their experiences, whereas others expressed themselves more briefly. The interviews were audio‐recorded and then transcribed using the Whisper software. All transcripts were subsequently reviewed to ensure accuracy and readability, and all identifying information was deleted from the transcripts.

This study is approved by the Swedish Ethical Review Authority (2025–01322, 2025–04558‐02). Informed consent was obtained from participants aged ≥ 15 years, or from all legal guardians if the participant was < 15 years of age. Participants aged < 15 years provided assent.

### 2.1 Data Analysis

Data were analysed in six phases using reflexive thematic analysis [13, 14]. To conduct the first phase, data familiarisation, we listened to the recorded interviews and reviewed the transcripts. Reflexive notes were taken while listening and reading. During this phase, reflexive notes captured a range of emotional and analytic reactions. EA and NMR both have personal experiences of bullying, which occasionally surfaced as a sense of recognition or emotional resonance when participants described their experiences. In the second phase, the data were coded in NVivo by EA and NMR. The reflexive notes shaped this process by encouraging a slower, more deliberate approach with frequent returns to the transcripts. Data were primarily coded using a semantic and inductive approach, focusing on the explicit rather than latent content and generating codes directly from the data without applying a pre‐existing theoretical framework. However, as acknowledged by Braun and Clarke [14], data can never be analysed without the influence of researchers' preunderstanding, and our understanding of weight‐related bullying was shaped by our awareness that obesity is a stigmatised disease [15, 16]. The initial coding generated 27 specific codes, such as “Responsibility is placed on the victim”, “You deserve this”, “School doesn't believe me”, and “Ignored”. The primary codes were subsequently integrated into broader codes. In the third phase, codes were further clustered to create candidate themes and subthemes such as “I can't escape this” and “I'm being punished for my body”. When organising codes into preliminary themes, we were attentive to the varied ways participants expressed themselves to avoid privileging more articulate accounts. We also reflected on how to weigh emotionally charged but less frequent codes, engaging in reflexive dialogue to ensure that our decisions were grounded in the dataset rather than shaped by emotional impact. Preliminary themes were critically reviewed together with KJ (a female clinical psychologist and associate professor with extensive experience in clinical work at a paediatric obesity clinic at a university hospital) to see if they were relevant for the aim of the study and captured the data coherently. While no major discrepancies were identified, moments of uncertainty helped refine the thematic structure. In phase four, the themes were further developed and refined by returning to the original transcripts to review the match between the themes and subthemes with the full data set. In the next phase, five, the themes were further refined by going back and forth between themes, codes, and transcripts in an iterative process to ensure that the themes represented a central organising concept. In this phase, the themes were given names. In phase six, the result section was drafted, and themes and subthemes were illustrated with quotes from the participants. Participant names in the results section are pseudonyms assigned by us, used to preserve the personal dimension of the data while ensuring confidentiality.

## 3 Results

The reflexive thematic analysis generated three themes, two of which comprised two subthemes each (Table 1).

**TABLE 1:** Themes and subthemes.

Themes	Subthemes
My body is the problem	The many faces of weight bullying
	When the body defines my value
When the school turns a blind eye	The school doesn't see me as a victim
	If you disappear, so does the problem
I'm ruined for life	

### 3.1 My Body Is the Problem

The first main theme, *My body is the problem*, captures how weight‐related bullying is experienced and enacted in everyday contexts, and how adolescents encounter pervasive messages devaluing their bodies and self‐worth.

#### 3.1.1 The Many Faces of Weight Bullying

Although all participants were, or had been, subjected to weight‐related bullying, its manifestations varied. A commonly reported experience among the adolescents, however, was receiving hurtful comments. Fatima shared that she was often called *“fatty”* and *“damn hippo”*. Emma told us that she was labelled “fat, ugly, and disgusting”, while Kim recalled hearing comments such as: “We should have Kim as the goalkeeper because she's so damn fat and covers the goal”, and that she was “a fatty who shouldn't even exist”. Sophia explained that she was always called *“fatty”* and that no one wanted to talk to her or play with her during breaks. All participants reported that these verbal insults could occur at any time. Kim noted that it could happen when she was heading out for break or going to the bathroom, whereas Maria described how the bullies often waited for her in the school canteen. Amina added that “there were always kids waiting somewhere”. In addition to derogatory comments about weight, participants described experiences of being assigned behaviours and negative traits stereotypically associated with obesity. For example, Ella explained that, because of her weight, people perceived her as “someone who likes McDonald's”, *“smells bad”*, and is *“generally disgusting”*.

Some participants also described forms of bullying that were more subtle, where weight‐related bullying was expressed through seemingly ordinary interactions but driven by underlying motives. Ella recounted such an experience: “There were like at least ten boys who said hi to me every day, like… sure, it can be nice to say hi, but they meant it in a very… not nice way, really.” Alex illustrated subtle bullying as follows: “They whispered about me as if I wouldn't notice. But I did anyway.”

Other participants spoke about physical bullying. Emma described experiences of violence associated with bullying during middle school: “It became more physical, and there was a lot of throwing erasers, throwing stones. Someone spat on me.” Amina also shared her experience of visible and violent bullying. When recounting her experience, she spoke in a matter‐of‐fact tone, as if presenting facts. Amina struggled to see how anyone without similar experiences could truly understand what this exposure has meant:

Bullying… it's easy to talk about and explain, but it's not easy to understand. Because it's like… the words aren't even a comma compared to how it really was. This happened daily. They spat in my food. They took my fruit and threw it on the ground. They dragged me into the bathroom and made me lick pee from the toilet. It was all of this… because I had a little extra fat on my body.

From the participants' narratives, it became evident that bullying was not confined to specific situations or locations within the school; rather, the harassment could occur virtually anytime and anywhere. Several participants reported that bullying sometimes took place publicly, in front of teachers and other students, yet few had experienced anyone standing up for them. Kim described it as follows: “It ends up being me who is the only witness, so to speak.” We interpret this as the participant being aware that others know the bullying is happening.

In summary, the participants' stories reveal the boundless nature of bullying, and the diversity of manifestations underscores its complexity and multidimensionality.

#### 3.1.2 When the Body Defines My Value

The relationship with one's own body is a theme that all participants touched upon during their interviews. They described how, from an early age, they developed a sense that something was wrong with their body. Alongside this early awareness, several participants described a simultaneous lack of understanding, as they were too young to grasp why others chose to focus on their bodies and evaluate them based on them. Sam explained to us: “I was quite little… how could I know what it means?” Emma reflected on the bullying she experienced in preschool and the confusion that arose when she sensed something was wrong but could neither explain nor understand what or why:

I became really sad, and I just couldn't understand what it was that… I felt that there was something terribly wrong with me, and I couldn't understand why. I didn't have any words to explain what was happening or words to explain why things were the way they were, because I didn't even understand it myself.

Other participants recalled the first time they realised they did not look like their classmates. This awareness was often triggered in moments when someone laughed, commented, or ridiculed them because of their weight. They spoke of feelings of sadness when they realised that what they initially perceived as a single, odd incident was, in fact, the beginning of what would become prolonged and systematic bullying. Ali described when his sense of belonging was replaced by exclusion: “I looked over at my classmates, and they were sitting there looking at me and whispering to each other, and then I thought, ‘now they've said something funny’… because I really thought they were my friends.”

Several participants described various attempts to hide their bodies as a way to avoid bullying. Emma shared that she wore a fleece sweater on her first day of preschool class (starts at age six) to cover her arms, despite it being mid‐August and warm outside. Similarly, Ella explained that she cannot remember the last time she wore short sleeves to school. Some participants also described how they changed their behaviour to protect themselves. Sophia recounted that she became so accustomed to hearing “stop eating, you're too fat” every time she ate something at school that she eventually stopped eating in front of others altogether to avoid the comments. Kim told us how everyday actions are perceived as risky, while at the same time she longs to be able to do them: “To dare to stand and cook in front of people. To eat the food at school. To dare to wear shorts one day when it's warm outside. There's so much you want.”

Some participants described how they clearly perceived that body weight, rather than other personal characteristics, was used by others as a basis for evaluating them as individuals and their actions. Ella described it as “your weight becomes your personality; it's how people see you”, and because the overweight body is attributed negative qualities, the entire individual is perceived as less desirable. She explained bluntly: “You are worth less than the skinny girl.”

When repeatedly confronted with messages that you are not more than your body, a body that is considered deviant and wrong, it may contribute to internalising the bullies' words as truth. Fatima said, “I had no friends because I was too fat”, and we interpret this as Fatima using the bullies' words as her own when explaining why she had no friends at school. Some participants also disclosed periods of self‐harm attributed to bullying. One participant shared that she harmed herself “because I had to get the pain out somehow… and it ended up being my own body.”

In summary, this subtheme illustrates how an early awareness of bodily difference, coupled with limited capacity to comprehend its social meaning, laid the foundation for prolonged stigma, which shaped both the participants' behaviours and identity.

### 3.2 When the School Turns a Blind Eye

This theme highlights the participants' experiences of the school environment and how the adults who should protect instead became part of the problem. The school, supposed to be a safe space, was for the participants transformed into a place marked by shame, loneliness, and resignation.

#### 3.2.1 The School Doesn't See Me as a Victim

All participants described how the school, in various ways, failed to recognise and act when they were subjected to bullying. A recurring experience concerned how school staff trivialised what was happening. Ali shared: “What was so hard was that the teachers didn't believe me either when I said I had been bullied.” Amina reported receiving no support regarding the bullying, despite teachers, principals, and teaching assistants being aware of what was going on. Not receiving any bullying‐related support, even though teachers both see and hear what is happening, is something all participants experienced. Charlie described this silence as follows: “It's like they… It's like it's right in front of them, but they don't see it. As if they pretend it's not happening. Yeah, it's like wearing sunglasses indoors.” In this way, bullying becomes not only something that occurs between students but something that is allowed to continue through the silence and passivity of adults. In some cases, bullying was even legitimised when school staff sided with the bullies. Emma recounted a situation where a bully said she was *“too fat”* to do a certain activity at school:

I was so incredibly hurt, and you know, everyone laughed, and then I remember I went to a teacher. I had really gathered my courage, and I was very upset, and I went to a teacher and told them, and then I got the response: ‘Well, he [the bully] is right about that.’

Some participants described how school staff downplayed their experiences by dismissing them as something all teenagers go through and suggesting that it was best to simply ignore what was happening. Fatima shared that the school used to say, “it's just everyday life”, when she reported the bullying. Several participants recounted that when they tried to stand up for themselves, the school told them not to respond, claiming that doing so would only make it more fun for the bullies.

Several participants also described situations where the school's response to bullying involved encouraging exercise or reducing food intake. Emma explained that the school used to say, “have a glass of water and sit down”, when she was hungry after one serving. She also shared how the school suggested her to run during lunch breaks and “try harder in PE [physical education]”. Emma reflected on the consequences of the teacher's comments: “That part also made you somehow believe that what you were subjected to was okay.” Charlie shared that the teacher initially taught all students to always take two servings of food, only to later tell him that he was no longer allowed to take two portions. When school staff encourage exercise or dietary restriction among bullied adolescents, this may be intended as support for obesity treatment. However, when bullied adolescents perceive it as implying that weight loss is necessary to stop the bullying, it reinforces self‐blame.

The participants' narratives reveal feelings of loneliness and abandonment. Amina described how she felt isolated both in relation to peers and teachers: “I was always singled out. Always left alone during breaks. Always had to sit and eat alone in the classroom. There was never a teacher who came over and wanted to sit with those who were alone.” Sam shared that when her previous school was confronted about its passivity, they referred to a lack of resources and said they did not have enough staff to provide support. At the same time, both Amina and Sam described brief but meaningful encounters with adults at school that made them feel momentarily seen, even if these moments did not alter the bullying itself. Amina shared that she remembers a teacher she could talk to when she first started school, and that this teacher is “still worth more than gold” to her. She explained that as they grew older, the teacher would not attempt to intervene when bullying occurred because “she can't shout at kids that are taller than she is.” Similarly, Sam recalled calling a teaching assistant for support after experiencing a panic attack at her current school: “He said he'd be three minutes, but he showed up after one minute! Then he took care of me and listened to me.” Sam explained that she had previously changed schools due to bullying and a lack of support, and although she is still being bullied, receiving this kind of care has started to make her feel safer at school. While the encounters described by Amina and Sam did not change the bullying they continued to experience, they highlight how simple acts of care can matter deeply to adolescents who otherwise feel unseen.

In this subtheme, the participants' accounts illustrate how the adolescent is left alone in their experience of themselves as a victim when the school fails to acknowledge the seriousness of the bullying. Blame and responsibility are shifted onto the adolescent, and the passivity of adults may contribute to the continuation of the bullying.

#### 3.2.2 If You Disappear, So Does the Problem

Several participants described school interventions aimed at removing them from situations where bullying occurred. Such strategies may signal that eliminating the victim's presence resolves the problem. Eventually, participants began to adopt withdrawal as a coping strategy when the situation became overwhelming.

Nicolas explained how the school's action plan involved isolating himself in a bathroom when bullying was happening, so that he could cry or rest, rather than receiving active support from school staff: “Yeah, it was just that I shouldn't be with them, and if they started teasing me… there's a bathroom by the principal's office, that's where I should go if they started teasing me and I felt tired and sad.”

In Natasha's case, the school decided she should change classes to make the bullying stop, and as a result, she was separated from all her friends. For several participants, PE classes were described as particularly difficult in relation to bullying. Kim shared that when this was raised with the school, they agreed that she would drop out of PE, and Ali explained that he either had to wait until everyone else had changed in the locker room or go and change in another room to avoid bullying after PE class. Removing the targeted adolescent thus not only signals that the solution is to disappear, but it also leads to the adolescent missing out on education and, as in Natasha's case, being isolated from friends.

Participants also began to adopt withdrawal as a coping strategy when situations became overwhelming. Several reported periods when they were unable to attend school. Kim explained: “During that time, I wasn't at school. I mean, you didn′t see me. I was at home crying.” Maria described how she tried to participate in PE classes as much as possible but would sit on the bench when older perpetrators entered the gym before their own lesson began:

And toward the end of PE, I was almost never involved. Like, when I saw that they started coming in, because they came in a little early, I sat down on the bench. And then I didn′t join the lesson. So I was really limited by them. And it was a big group of boys, almost all the boys in that class.

Absence and non‐participation become a form of protection, but also an exclusion, one that the school, paradoxically, has endorsed.

In contrast to the social withdrawal described by several participants, some adolescents described using emotional withdrawal to cope with the bullying. Sophia said, “I just try to pretend that I don′t hear them. So maybe that's where I've found a small solution.” Similarly, Natasha shared: “I′ve kind of taught myself to just switch off my brain”, while Ali “eventually just learned to ignore them completely.” These strategies further illustrate how the adolescents try to protect themselves when the bullying persists.

Rather than escape, the participants asked for recognition. All participants wished that the school had listened to them and validated their experiences, and Emma said: “I think that, first and foremost, I just would have wanted to hear that what you're going through is not okay.” Fatima shared that she wished school staff had told the bullies: “This is bullying, what you′re doing”, since the school never recognised what was happening as bullying. Amina expressed that she wished school staff “had listened, let us speak, and taken our words seriously.” Natasha said she wished the school had asked “How are you?” more often. Similarly, Maria explained that the school never asked her why she was alone or staying in the classroom during breaks, and that she would have wanted someone from the staff to approach her and talk to her. Emma reflected on responsibility and her wishes: “Teachers' power may be limited, but somehow it felt wrong when they said that I should change groups, or that I should avoid them… and that's where I wish it had been more the other way around.”

This subtheme reflects participants' perceptions that schools primarily relied on interventions aimed at removing victims from bullying situations, while participants expressed a clear need for recognition and support. Over time, participants also began using avoidance and withdrawal to escape the bullying.

### 3.3 I′m Ruined for Life

This theme illustrates how the adolescents describe bullying as gradually breaking them down. In the absence of support from adults, feelings of hopelessness and mistrust emerge, which in some cases prevent the adolescent from daring to dream or believe in their own future.

All participants described how attending school became difficult or even impossible during certain periods, and several participants described how they stopped reporting weight‐related bullying to the school because they did not trust the school to make a difference. Sophia explained why she no longer reports incidents: “…because I know they won't help with anything.” Nicolas was asked whether he felt the support he received from the school was sufficient: “Well, they've told them [the bullies] many times, but they don't listen… so yeah, they did what they could do, so… yeah.” Thus, Nicolas expresses some kind of acceptance, yet his words are marked by resignation. Within this resignation lies an awareness that the school should have been there to protect him. Kim also described her resignation and lack of hope in relation to the school:

And it really took a lot out of me. That nothing… nothing happened. And it became very mentally exhausting, too. That… no one takes me seriously. Not even the school does. So then, in the end, no one will take me seriously. And it will just end up with me not being able to cope with this anymore.

The participants' narratives also revealed how their trust in others more generally had been negatively affected by the bullying. Alex shared that she did not want to go to school on some days, and when she did attend, she actively tried to avoid conflicts with her friends because she feared they might side with the bullies. Similarly, Sophia expressed how her trust in friends had been affected, explaining that she does not trust them enough to confide in them about the bullying. For Ella, the bullying experiences had led to a distrust of most people. She stated, “Many people who are nice to me aren't genuine, and that's just something I've learned to accept these days.” Likewise, Maria shared that she often feels discomfort when meeting new people, because “as soon as they whisper or something like that, I think it's about me, because that's how it was before.” This mistrust could be understood as a form of self‐protection aimed at reducing the risk of new disappointments, while at the same time potentially hindering the creation of new relationships.

For many, being bullied in school resulted in a fear of being victimised also in other settings. Emma revealed that today she has “an incredible fear of public spaces” because of her experiences. Other participants described feeling uneasy about being out in public with their families, in case someone might shout something hurtful or throw glances. Ella explained that she actively tries to cover her body when she is out in public “because it's better for everyone not to see”. We hear a sense of hopelessness in Ella's words, as if she is certain about what will happen if she does not cover her body. For the victimised adolescents, the public spaces no longer appear neutral, but rather as places of fear and shame.

Weight‐related bullying was not seen as only affecting the present but also the future. Ali expressed: “There are certain words people have said to me that will stay in my head for life”, which can be understood as a fear of never escaping the pain that the bullying has caused. Being trapped in that pain and unable to dream about the future is something several adolescents described. Kim articulated it as follows:

I find it hard to see myself later in life. I find it hard to see myself with children… because this has happened. I find it hard to see that I'll manage to live a normal life, with children, family. Like family, a house and pets. And being able to travel. All those things you're supposed to be able to do. And to be able to dream about. But I can't dream about it, because I don't know what my future will look like. And if I'll get that far in my future, if I'll have the strength to keep going and make it that far in my life.

This third main theme illustrates the adolescents' experiences of weight‐related bullying as a profoundly damaging process that erodes trust in others and inflicts lasting harm.

## 4 Discussion

This qualitative study explored adolescents' experiences of weight‐related bullying within the school context and their perceptions of the school's strategies for addressing it, as well as the measures they wished had been implemented. Reflexive thematic analysis generated three themes and four subthemes. Together, these themes illustrate the extent to which adolescents were victimised because of their weight and how they perceived its impact on their well‐being, self‐concept, and expectations regarding relationships and the future. The findings also reveal that adolescents felt abandoned by school staff when bullying was not taken seriously, when efforts to stop it failed, or when attention shifted to their weight rather than to the perpetrators.

This study adds to the limited literature about weight‐related bullying in the school context by giving voice to exposed adolescents. The analysis of their stories provides a deeper understanding of their lived experience. Previous quantitative studies have shown that bullying is associated with an increased risk of severe mental health problems, including substance abuse and suicide [4, 17, 18]. Qualitative research can help generate hypotheses regarding the mechanisms underlying these associations. Participants in the present study spoke not only about the pain caused by the bullying itself but also about the harm they experienced from the schools' inability to protect them. These findings suggest that it is important to examine whether effective school‐based anti‐bullying interventions can reduce the negative psychosocial consequences associated with weight‐related bullying.

Weight‐related bullying in school is associated with school absenteeism [8, 19], and the participants described that both the school and themselves used exclusion and withdrawal as a way of coping with the bullying. Research shows that children and adolescents with obesity finish their education to a lesser degree than their peers, regardless of socioeconomic status [20]. Future studies should explore the extent to which weight‐related bullying explains school dropout and incomplete education in adolescents with obesity.

The findings have implications for both health care providers and professionals working within the school setting. The participants' narratives underscore the profound distress caused by weight‐related bullying, and all clinicians treating children and adolescents with obesity should be well‐informed about its prevalence and routinely ask patients about their school environment and whether they experience such bullying. If a child or adolescent reports being bullied, and the family perceives that the school is not taking adequate measures to stop it, clinicians should offer to communicate with the school and emphasise the serious negative impact bullying has on young people's current and future health [3, 4, 5, 6, 7, 8, 9].

Another important finding is that the adolescents' narratives reveal a recurring pattern of self‐imposed restrictions aimed at enduring their circumstances and avoiding further humiliation, such as concealing their bodies and refraining from eating in the presence of others. For clinicians meeting adolescents with obesity, it can be important to understand that a generalised avoidance behaviour may have originated as a coping strategy to protect against bullying [21, 22]. Self‐imposed restrictions can also be understood as internalised weight stigma [23, 24], where the words and actions of bullies have been incorporated into the self‐concept. More frequent exposure to weight stigma, such as bullying, is shown to result in more disengagement forms of coping, which in turn are related to poorer psychological well‐being [23].

Frequent exposure to weight stigma increases the likelihood of internalising weight‐related stereotypes and beliefs [23]. Adolescents in our study described how their body weight was repeatedly used by others as a basis for evaluating them as individuals. Such experiences can be particularly detrimental during self‐identity development, when young people are forming a coherent sense of who they are in relation to others [25]. When repeatedly confronted with messages that their body defines their value, adolescents may come to place disproportionate importance on weight and shape when evaluating their self‐worth. Given that weight‐related bullying, internalised stigma, and overvaluation of weight and shape are associated with disordered eating [7, 16, 26], it can also be important for clinicians to be attentive to changes in eating patterns that could indicate emerging maladaptive coping responses.

The adolescents in our study described a sense that the school did not take weight‐related bullying as seriously as other forms of bullying. This is in line with findings showing that weight stigmatisation is tolerated in society based on false beliefs that it will motivate people to lose weight [27]. This study was conducted in Sweden, and although weight‐related bullying is the most common form of bullying [2, 28], it is not mentioned in governmental reports intended to address discrimination, bullying, and other degrading treatment in Swedish schools [29]. Nevertheless, shortcomings in addressing weight‐based bullying are not unique to Sweden. Evidence from a study conducted in England indicated that only 6.7% of schools explicitly referenced weight‐based bullying in their anti‐bullying policies, compared to 94.5% and 93.3% for race/ethnicity and sexual orientation, respectively [30]. Schools should recognise weight‐related bullying as equally serious as other forms of bullying and explicitly include it in their action plans. Research has shown that explicitly mentioning weight in anti‐bullying policies reduces educators' explicit weight bias [31].

Some participants perceived the school's focus on altering their diet or increasing their exercise as an attempt to make the bullying end, which reinforced the feeling that the problem lay with their own body and that the bullying would cease only if the body changed. It is plausible that this was not the school's intended message, and their aim may have been to support weight management irrespective of the student's social context. Nevertheless, a key finding of the present study is that schools must unequivocally communicate to students bullied for their weight that the responsibility for change lies with the perpetrators, not the victims.

Strengths of the study include providing a rich, first‐hand perspective on how weight‐related bullying is experienced by adolescents. An additional strength is that participants were still of school age, allowing insights into current experiences rather than retrospective accounts. The inclusion of both boys and girls adds gender diversity to the sample, and the relatively long interviews generated a nuanced and comprehensive dataset.

During participant recruitment, many adolescents reported that they were or had been subjected to bullying but did not want to talk about their experiences. Studies like this can only reflect the experiences of those adolescents who are willing to be interviewed, and we may have missed important experiences from those who did not want to take part. As recruitment was primarily conducted via obesity clinics and the patient association Obesity Sweden, participants were drawn from contexts in which obesity is framed as a medical condition and where the term itself is generally accepted and well understood. This recruitment strategy may have excluded individuals with higher body weight outside such contexts, for whom the term obesity may be experienced as stigmatising or contested. The study was conducted among Swedish adolescents, and it is unknown to what extent the findings are transferable to adolescents in other countries with more developed anti‐bullying strategies related to weight [31].

## 5 Conclusion

When adolescents with obesity were interviewed about their experiences of weight‐related bullying, they described how both explicit and subtle forms of bullying reduced them to their weight and bodies, causing harm to their relationships with themselves and others, both now and in the future. They felt betrayed by the school, which did not take the bullying seriously enough and failed to stop it. Those who interact with adolescents with obesity, whether as clinicians or within the school setting, need to understand the damage that bullying causes, be prepared to listen to the adolescents' experiences and take them seriously, and do everything in their power to ensure that no child or adolescent is subjected to bullying because of their weight.

## Author Contributions

**Emelie Andersson:** investigation, data curation, writing – original draft, formal analysis, project administration, software. **Kajsa Järvholm:** conceptualization, methodology, validation, writing – review and editing, supervision. **Nicole Mpatsiaris Riboe:** writing – original draft, investigation, data curation, formal analysis, project administration, software.

## Funding

The authors have nothing to report.

## Conflicts of Interest

K.J. has received speaker honoraria from Novo Nordisk, unrelated to this study and directed to her clinical institution (Region Skåne). The other authors have no conflicts of interest to declare.

## Data Availability

The data that support the findings of this study are available on request from the corresponding author. The data are not publicly available due to privacy or ethical restrictions.
